# Ginkgetin from *Ginkgo biloba*: mechanistic insights into anticancer efficacy

**DOI:** 10.1007/s13659-025-00535-6

**Published:** 2025-08-05

**Authors:** Bei Xiong, Jin-Jian Lu, Hongwei Guo, Mingqing Huang, Ting Li

**Affiliations:** 1 State Key Laboratory of Quality Research in Chinese Medicine, Institute of Chinese Medical Sciences, University of Macau, Macao SAR, 999078 China; 2 MoE Frontiers Science Center for Precision Oncology, University of Macau, Macao SAR, 999078 China; 3 Department of Pharmaceutical Sciences, Faculty of Health Sciences, University of Macau, Macao SAR, 999078 China; 4 Guangxi Key Laboratory of Bioactive Molecules Research and Evaluation & Pharmaceutical College, Guangxi Medical University, Nanning, 530021 China; 5 College of Pharmacy, Fujian University of Traditional Chinese Medicine, Fuzhou, 350108 China

**Keywords:** Ginkgetin, Ginkgo biloba, Biflavones, Natural product, Anti-cancer, Mechanism

## Abstract

The extraction of anticancer agents from medicinal plants represents a highly promising research frontier. Ginkgetin, a natural biflavone, is one of the effective pharmacological components of *Ginkgo biloba* leaves (GBLs). This natural product exhibits significant anti-cancer efficacy against a variety of cancer cells in vitro and demonstrates a potent inhibitory impact on tumor growth in vivo without severe toxicity. Additionally, ginkgetin synergizes with chemotherapy drugs or adjuvant therapies to potentiate antitumor effects and reduce side effects. These compelling findings underscore Ginkgetin's potential as a promising candidate for novel anti-cancer therapeutics. Therefore, this review systematically summarizes the remarkable anticancer effects of ginkgetin and elucidates its multifaceted anticancer mechanisms, including inducing cell cycle arrest, triggering programmed cell death, and preventing invasion and angiogenesis. From a molecular mechanism perspective, ginkgetin exerts anti-cancer activity by modulating critical signaling pathways (e.g. JAK/STAT, Wnt/β-catenin, AKT/GSK-3β, MAPKs, and estrogen receptor pathways) and regulating microRNA expression levels. Furthermore, target identification, research limitations, future directions, and application prospects are comprehensively outlined, aiming to facilitate the clinical translation of ginkgetin.

**Graphical Abstract:**

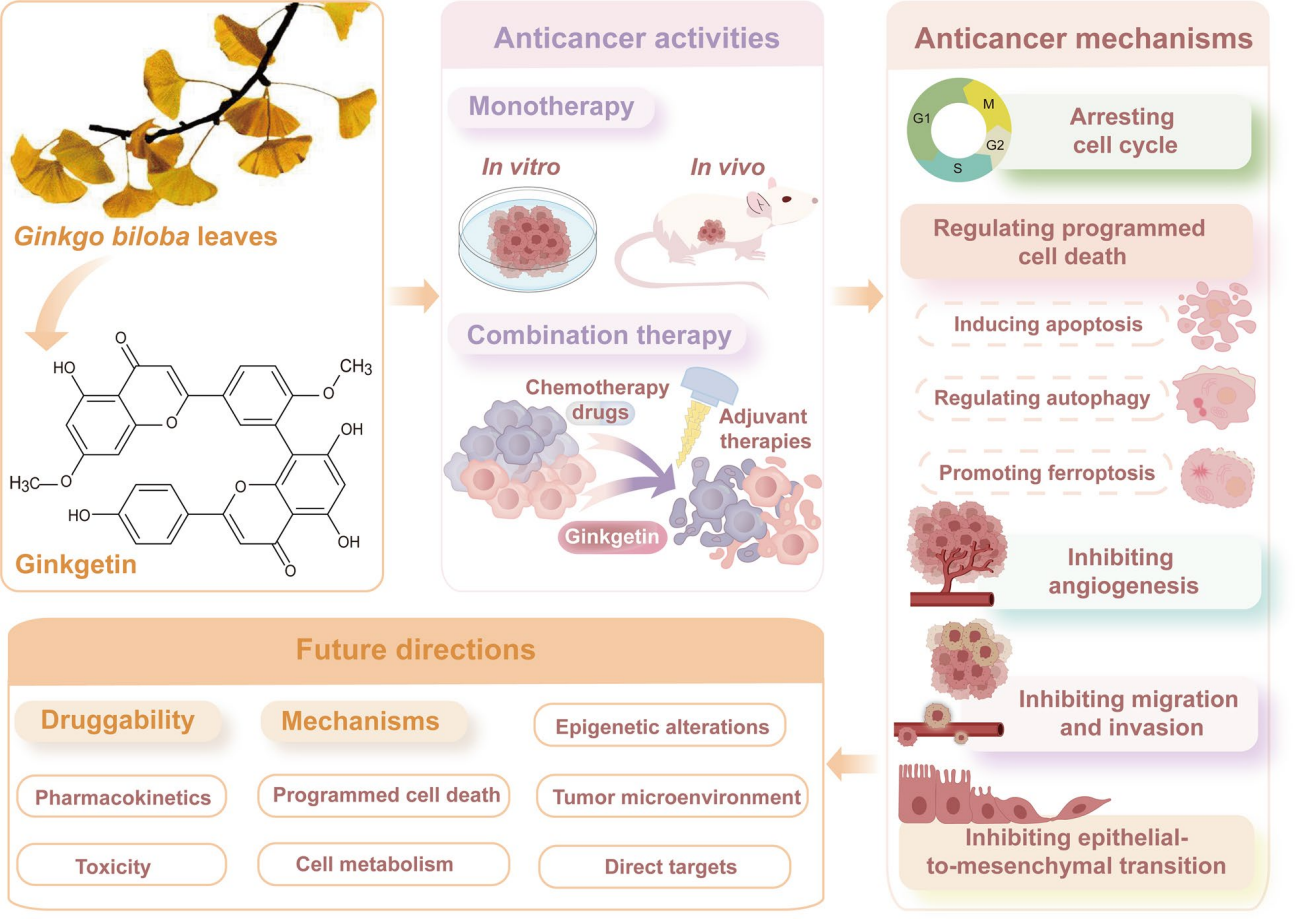

## Introduction

*Ginkgo biloba* is one of the oldest living organisms on Earth, known as a “living fossil” [1, 2]. Originating from China, it has gained global distribution owing to its significant medicinal properties and ornamental appeal [3]. *Ginkgo biloba* leaves (GBLs) have been used as herbal medicine to treat diseases for thousands of years in China, Japan, and South Korea [4]. According to Essentials of Materia Medica Distinctions, GBLs have the effects of relieving cough, resolving phlegm, and restoring lung function [5]. In the 1960s, European countries began to pay attention to the research of GBLs extracts [6]. EGb 761, which contains 24% of ginkgo flavone glycosides and 6% of terpene trilactones, is regarded as the proprietary, standardized extract obtained from GBLs [7, 8]. Due to its safety and efficacy, GBLs have become one of the most widely used herbs in Europe and the United States, with annual sales of related products reaching billions of dollars [9, 10]. Nowadays, GBLs extract formulations have been used clinically for the treatment of cardiovascular and cerebrovascular diseases, as well as elderly dementia and other diseases [11–13]. In addition, an increasing number of studies have demonstrated that GBLs and their components exhibit significant anti-cancer properties through multifaceted mechanisms such as inhibition of angiogenesis, modulation of gene expression and so on [14]. The therapeutic applications of GBLs in oncology represent an emerging and promising research domain.

**Fig. 1:**
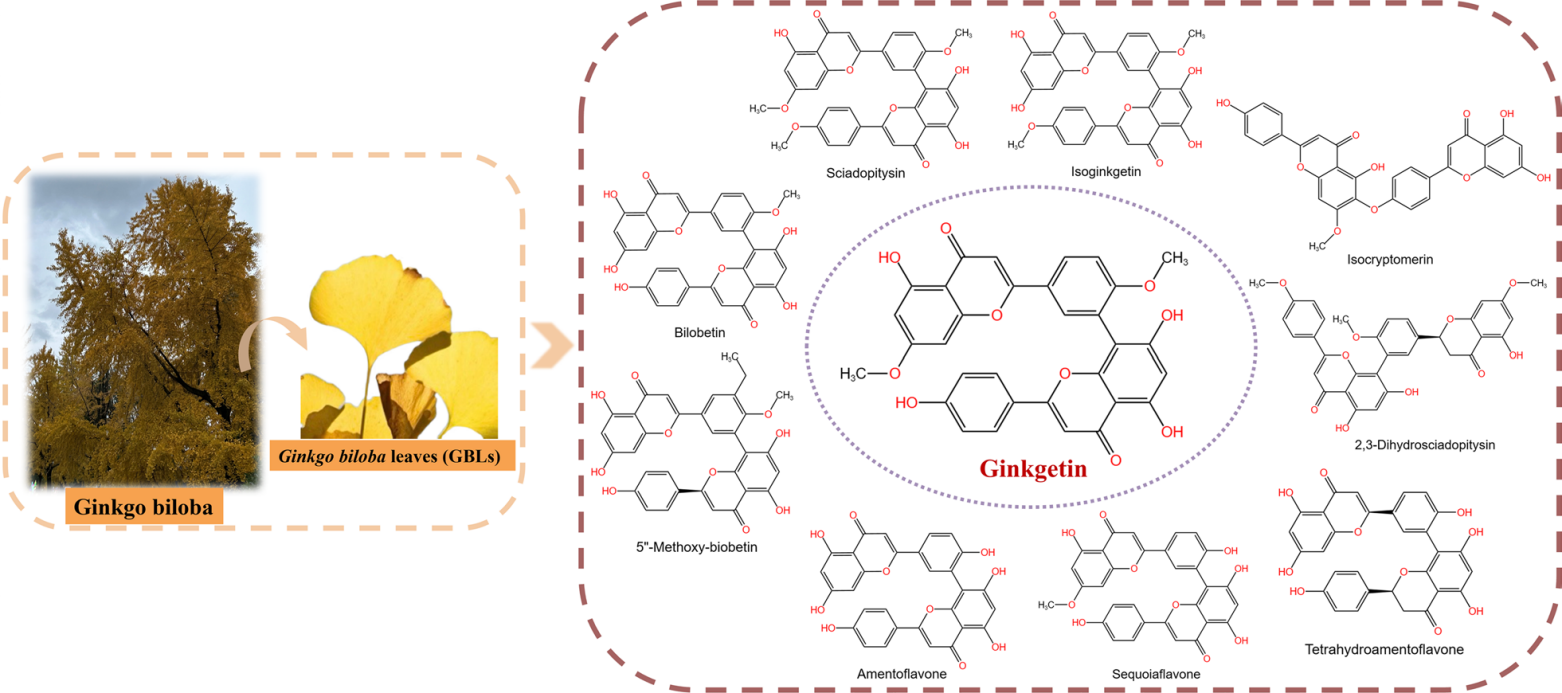
Chemical structures of biflavones isolated from Ginkgo biloba leaves. Including Sciadopitysin, Isoginkgetin, Isocryptomenin, 2,3-Dihydrosciadopitysin, Tetrahydroamentoflavone, Sequoiaflavone, Amentoflavone, 5″-Methoxy-biobetin, Bilobetin, and Ginkgetin

Flavonoids and terpene lactones constitute the primary bioactive constituents in GBLs, with mono- flavonoids and biflavonoids representing the predominant active fraction [15]. To date, over thirty mono-flavonoids, including quercetin, kaempferol, and isorhamnetin, have been isolated from GBLs [16]. Biflavones, as a distinct category of flavonoids, are dimeric structures formed by two mono-flavone units. They exhibit notably higher bioactivity than mono-flavones in specific pharmacological contexts [17]. Currently, 10 types of biflavones (Fig. 1) have been identified in GBLs, with ginkgetin (33.79 ± 2.80 µg/g dry weight) being the most abundant [18, 19]. Since ginkgetin was isolated as a standalone compound in 1996, research reports on its biological activity have continuously emerged, including anti-inflammatory [20, 21], anti-bacterial [22, 23], anti-fungal [24, 25], anti-virus [26], anti-atherosclerosis [27, 28], anti-adipogenesis [29], anti-oxidant [30, 31], anti-senescence [32, 33], anti-fibrotic [34, 35] and neuroprotective [36, 37]. Moreover, emerging evidence suggests that ginkgetin could enhance the body’s immunity and has an anti-tumor function [38, 39]. Ginkgetin has shown anti-cancer effects in vitro and in vivo in many cancers, including lung cancer, ovarian cancer, breast cancer, prostate cancer, colon cancer, and leukemia [40]. Accumulating evidence indicates that ginkgetin elicits its anti-tumor efficacy through multifaceted molecular mechanisms rather than a singular pathway. These mechanisms involve inducing cell cycle arrest, triggering programmed cell death, preventing migration and invasion, and inhibiting angiogenesis. Ginkgetin can also be used in combination with other drugs and types of treatment to improve treatment effectiveness and reduce side effects. Therefore, ginkgetin warrants further in-depth investigation to validate its potential as a viable and effective anti-cancer therapeutic agent.

Focusing on ginkgetin’s broad anticancer potential, the discussion surveys its molecular underpinnings to inform compound optimization, therapeutic deployment, and future oncology studies, while also outlining the critical research needed to bolster druggability, clarify mechanistic action, and authenticate molecular targets for successful clinical translation.

## Methods

This review article summarized the literature published before May 2025. Relevant articles were search on electronic academic databases such as Web of Science, PubMed, Scopus, Google Scholar, and Chinese National Knowledge Infrastructure using keywords including ginkgetin, biflavones, cancer, target, pharmacokinetics, and toxicity. An overall evaluation was conduct of the 269 retrieved articles, removing those that are duplicate, irrelevant, have unclear information and contain obvious errors. Subsequently, the remaining articles were classified and summarized to provide an overview of the research advancements in the anticancer potential of ginkgetin. Finally, in the discussion section, the anti-cancer efficacy and mechanisms of current research on ginkgetin were summarized, and shortcomings in research and future research directions were also proposed.

## Anticancer activities

### Monotherapy

#### Anticancer activities in vitro

Ginkgetin has been demonstrated to possess anti-tumor activity on several types of cancer cells, including breast cancer [41–44], cervical cancer [45, 46], colon cancer [47, 48], hepatocellular carcinoma [49], kidney cancer [50], leukemia [51], lung cancer [52–55], medulloblastoma [56], myeloma [57], neck cancer [52], osteosarcoma [58], ovarian cancer [39, 45, 59], and prostate cancer [38, 60]. When treated with ginkgetin in vitro for 24 to 96 h, the IC_50_ values for inhibiting cancer cell proliferation ranged from 0.58 to 150 μM, as more details are presented in Table 1. Ginkgetin inhibits the proliferation of most cancer cells at low concentrations, with IC_50_ values below 5 μM when administered for 24 h or longer in various cancer types, including colon cancer, lung cancer, myeloma, ovarian cancer, prostate cancer, and so on. Additionally, within the same cancer cell line, ginkgetin exerts its anti-cancer effects in a time- and dose-dependent manner, such as in breast cancer, colon cancer, leukemia, lung cancer, etc. The magnitude of the IC_50_ value reflects the relative sensitivity of cancer cells to ginkgetin to some extent. However, it is noteworthy that differences in experimental conditions, such as cell density, culture medium composition, and detection methods, lead to variations in the measurement of IC_50_ values. The varying sensitivities of different cancer cell lines to ginkgetin may be attributed to differences in the expression levels of ginkgetin-binding targets and the activity levels of associated signaling pathways across these cells.

**Table 1:** Antiproliferative activities of ginkgetin and molecular targets in vitro

Cancer type	Cell line	IC_50_ (μM)	Time (h)	Assay	Refs.
Breast cancer	MCF-7	10	24	MTT	[41]
	T-47D	10			
	MDA-MB-231	150; 81; 32; 18	24; 48; 72; 96	MTT	[42]
	BT-474	/; 150; 44; 32			
	MCF-7	80; 60; 40; 26			
	MDA-MB-231	11.5; 9.302	48; 72	CCK-8	[44]
	MDA-MB-453	5.699; 5.545			
	MDA-MB-436	10.88; 3.707			
	MCF-7	8.315; 6.736			
	SUM-159	5.653; 3.916			
	4T1	8.054; 7.397			
	4T1	4.34	24	CCK-8	[43]
Cervical cancer	HeLa	5.2	48	MTT	[45]
	HeLa	/	48	MTT	[46]
Colon cancer	HCT116	4	48	WST-1	[47]
	SW620	3.5			
	HCA7	10			
	HCT116	0.58	72	Resazurin	[48]
	RKO	0.75			
	LOVO	0.6			
Hepatocellular carcinoma	HepG2	/; 45	24; 48	MTT	[49]
	SK-HEP-1	/; 38			
Kidney cancer	786-O	7.23	48	MTT	[50]
Leukemia	K562	38.9; 31.3; 19.2	24; 48; 72	MTT	[51]
Lung cancer	A549	/	24	MTT	[52]
	H1299	2.789			
	A549	10.16; 5.21; 2.73	24; 48; 72	MTT	[54]
	PC9	16.98;11.02; 5.78			
	NCIH-460	23.17; 7.76; 4.98			
Medulloblastoma	Daoy	14.65 ± 0.07	48	MTT	[56]
	D283	15.81 ± 0.57			
Myeloma	RPMI-8226	3.5	48	CCK-8	[57]
Neck cancer	FaDu	/	24	MTT	[52]
Osteosarcoma	/	35.5	24	MTT	[58]
Ovarian cancer	OVCAR-3	1.8	24	MTT	[39, 45]
	A2780	11.46	24	MTT	[59]
	SK-OV-3	12.18			
	CP70	17.84			
Prostate cancer	DU145	5	48	CCK-8	[38]
	PC-3	40	24	MTT	[60]

#### Anticancer activities in vivo

Ginkgetin treatments through intraperitoneal injections (*i.p.*) or oral administration (*p.o.*) remarkably repressed tumor growth in mouse xenograft models without notable declines in body weight and visible toxicity. The concentration for *p.o.* was between 20 and 100 mg/kg, while the concentration for *i.p.* was between 10 and 30 mg/kg. The tumor inhibition rate was approximately between 20 and 70%, and *i.p.* was more effective. For instance, *i.p.* of 30 mg/kg ginkgetin five times a week reduced the volume and weight of DU145 xenograft tumors in nu/nu mice by 65.6 and 67.4%, respectively [38]. Furthermore, ginkgetin also suppressed tumor metastasis [53]. In the lung metastasis model established by injecting LLC cells into the tail vein of mice, after daily *i.p.* of ginkgetin (15 or 30 mg/kg) for 2 weeks, the number of lung tumor nodules was approximately 60 and 20% of the control group, respectively [53]. Additional details are shown in Table 2.

**Table 2:** Anticancer activities of Ginkgo biloba leaf extracts in vivo

Cancer type	Animal models	Cell line	Dose	Administration time	Administration route	Effects	Refs.
Breast cancer	BALB/c nude mice	MCF-7	25 mg/kg	Daily for eight weeks	*p.o*	Tumor inhibitory rates were about 20%, 30%, 50%	[42]
			50 mg/kg				
			100 mg/kg				
	SCID	MDA-MB-231	30 mg/kg	Five days per week for six weeks	*i.p*	Tumor inhibitory rates were about 35%	[44]
Colon cancer	BALB/c nude mice	HCT116	10 mg/kg	Five times per week for 23 days	*i.p*	Causes a 36.5% decrease in tumor volume and a 37.6% decrease in tumor weight	[47]
	BALB/c nude mice	HCT116	10 mg/kg	Daily for 12 days	*i.p*	Tumor inhibitory rate was about 40%	[48]
Leukemia	BALB/c nude mice	K562	20 mg/kg	Daily for 2 weeks	*p.o*	Tumor inhibitory rate was about 40%	[51]
Lung cancer	C57BL/6 wild-type mice	LCC	15 mg/kg	Daily for 2 weeks	*i.p*	Suppressed lung metastasis to about 60 and 20%	[53]
			30 mg / kg				
	BALB/Cnude mice	A549	30 mg/kg	Daily for 40 days	*i.p*	Tumor inhibitory rate was 50%	[54]
Ovarian cancer	xenograft nude mouse	A2780	30 mg/kg	Daily for 19 days	*p.o*	Tumor inhibitory rates were about 50% and 60%	[59]
			45 mg/kg				
Prostate cancer	BALB/c nude mice	Du145	30 mg/kg	Five times per week for 23 days	*i.p*	Reduces tumor volume by 65.6% and tumor weight by 67.4%	[38]

### Combination therapy

In addition to single treatment, ginkgetin has a synergistic effect when used in combination with some other drugs and types of treatment, as shown in Table 3. When used in combination with cisplatin, it promoted cisplatin-induced cytotoxicity in vitro [61]. In vivo, the tumor weight in the combination therapy group was only about 50% of that in the cisplatin group, with an increase in body weight [61]. In addition, ginkgetin and resveratrol had a synergistic effect in suppressing VEGF-induced angiogenesis [62]. In HT-29 colon cancer xenograft nude mice, the combination of ginkgetin and resveratrol synergized the anti-tumor effect of 5-Fluorouracil (5-FU) by reducing tumor microvascular density. The tumor inhibition rate of the 5-FU monotherapy group was approximately 30%, while the tumor inhibition rate of 5-FU plus the combined ginkgetin-resveratrol at high dose was 55% [62]. Furthermore, ginkgetin alone also enhanced the anti-tumor effect of 5-FU in HCT-116 cells and HCT-116 colon cancer xenograft models [48]. In addition to being used in combination with drugs, ginkgetin combined radiotherapy suppressed breast tumor growth without inducing liver damage, demonstrating a synergistic effect between radiation and ginkgetin in tumor tissues [43].

**Table 3:** The in vivo anticancer effects of ginkgetin combined with other drugs or therapy

Cancer type	Animal models	Cell line	Mode of administration	Tumor inhibitory rate (%)	Refs.
Breast cancer	BALB/c mice	4T1	Ginkgetin (5 mg/kg, daily for 7 days,* i.p.*)	50	[43]
			Irradiated (8 Gy, daily for 7 days)	55	
			Ginkgetin (5 mg/kg, daily for 7 days,* i.p.*) + Irradiated (8 Gy)	87.5	
Colon cancer	BALB/c nude mice	HCT-116	Ginkgetin (10 mg/kg, daily for 12 days,* i.p.*)	45	[48]
			5-FU (30 mg/kg, once every 3 days for 12 days,* i.p.*)	33	
			Ginkgetin (10 mg/kg, daily for 12 days,* i.p.*) + 5-FU (30 mg/kg, once every 3 days for 12 days, *i.p.*)	78	
	BALB/C nude mice	HT29	5-FU (30 mg/kg, once every 2 days for 30 days,* i.p.*)	30	[62]
			Ginkgetin (800 mg/kg, daily for 30 days,* i.g.*) + 5-FU (30 mg/kg, once every 2 days for 30 days,* i.p.*)	49	
			Ginkgetin (80 mg/kg, daily for 30 days,* i.g.*) + resveratrol (240 mg/kg, daily for 30 days,* i.g.*) + 5-FU (30 mg/kg, once every 2 days for 30 days, *i.p.*)	50	
			Ginkgetin (160 mg/kg, daily for 30 days,* i.g.*) + resveratrol (480 mg/kg, daily for 30 days,* i.g.*) + 5-FU (30 mg/kg, once every 2 days for 30 days,* i.p.*)	52	
			Ginkgetin (320 mg/kg, daily for 30 days,* i.g.*) + resveratrol (960 mg/kg, daily for 30 days,* i.g.*) + 5-FU (30 mg/kg, once every 2 days for 30 days,* i.p.*)	55	
Lung cancer	Nude mice	A549	Ginkgetin (30 mg/kg, daily for 32 days,* i.p.*)	46	[61]
			Cisplatin (3 mg/kg, 2—3 times per week for 32 days,* i.p.*)	56	
			Ginkgetin (30 mg/kg, daily for 32 days,* i.p.*) + cisplatin (3 mg/kg, 2—3 times per week for 32 days,* i.p.*)	75	

## Anticancer mechanisms

### Arresting cell cycle

Uncontrolled cell division is a hallmark of cancer cells [63]. Arresting cell cycle can prevent dysregulated cell division and hinder tumor growth. Ginkgetin at a concentration of 5 μM caused accumulation of prostate cancer cells in the G0/G1 phase of the cell cycle in a time-dependent manner, 55.5% of cells were in the G0/G1 phase after 9 h treatment [38]. In addition, ginkgetin within the concentration range of 10 to 30 μM induced G2/M cell cycle arrest in medulloblastoma and colon cancer cells [47, 56]. For example, the percentage of HCT116 cells in G2/M phase increased by 2.2-fold (43.25%) versus the untreated control (19.69%) when treated with 10 μM ginkgetin for 48 h [47]. Furthermore, ginkgetin arrested human hepatocellular carcinoma cell at the S phase in a dose- and time-dependent manner [49]. Ginkgetin induces cell cycle arrest at distinct phases in various tumor cell types, potentially mediated by its differential engagement with cell type-specific signaling pathways or regulatory networks. Additionally, there are differences in cell cycle length and characteristics between different cell lines, and the dosage and treatment time of ginkgetin also affect the phase of cell cycle arrest.

**Fig.2:**
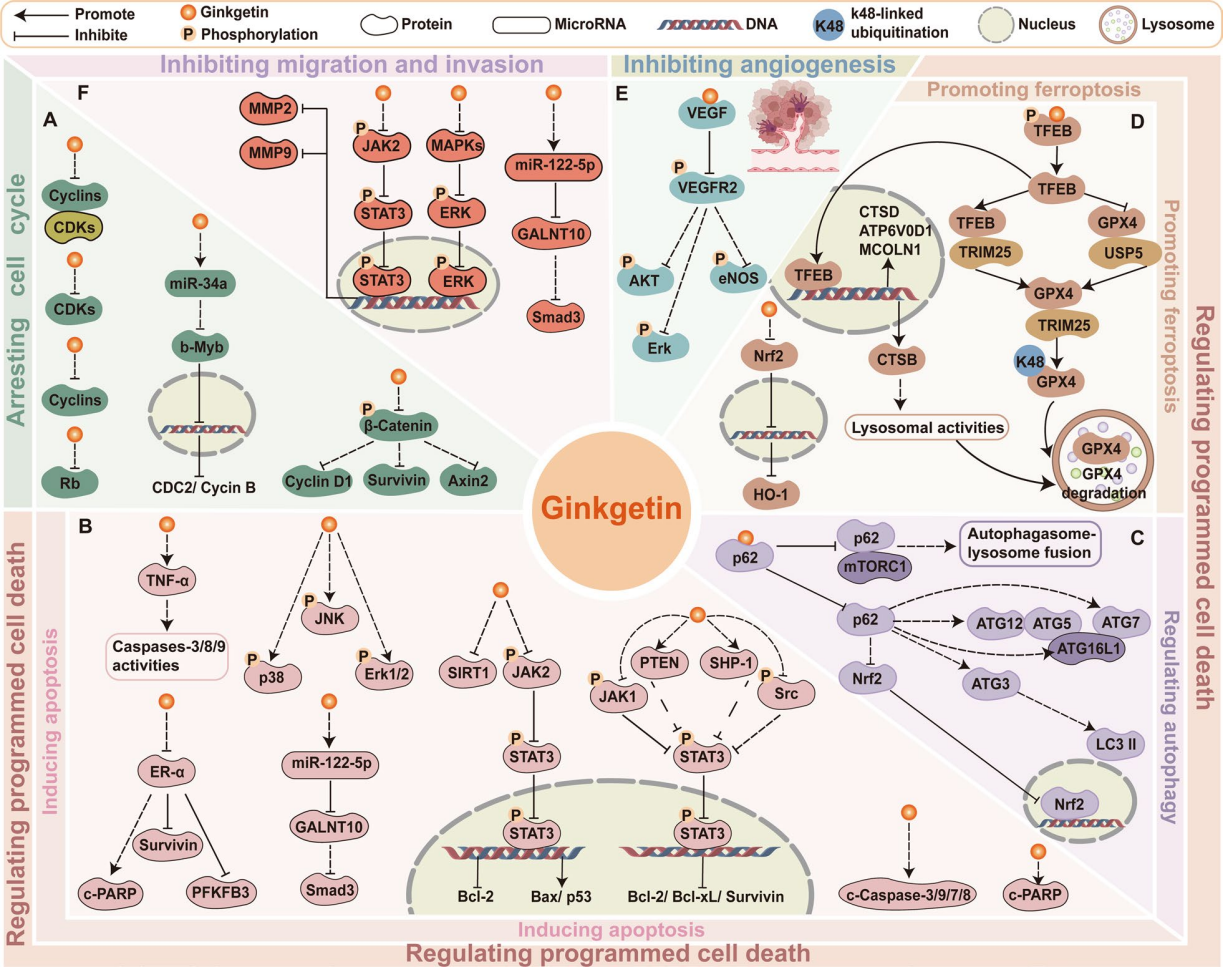
Schematic representation of ginkgetin’s partial anticancer mechanisms. **A** Arresting cell cycle. **B** Inducing apoptosis. **C** Regulating autophagy. **D** Promoting ferroptosis. **E** Inhibiting angiogenesis. **F** Inhibiting migration and invasion. The regulatory mechanisms of selected relevant molecules are depicted in the figure

Forming essential regulatory complexes, cyclins and cyclin-dependent kinases (CDKs) exert pivotal control over cell cycle progression, with their frequent dysregulation being a hallmark of malignant cells [64]. Ginkgetin effectively modulated the expression of cyclins and CDKs to arrest the cell cycle. Ginkgetin treatment led to the downregulation of cyclin D1 in prostate cancer cells, osteosarcoma cells, and medulloblastoma cells [38, 56, 58]. Specifically, ginkgetin was an inhibitor of Wnt signaling, reduced the expression of Wnt target genes cyclinD1, survivin, and Axin2 in medulloblastoma cells [56]. Besides, in HCT-116 colon cancer cells, ginkgetin-mediated microRNA modulation targeted the miRNA34a/b-Myb/cyclin B1 cascade, resulting in G2 phase arrest through downstream regulation of b-Myb, CDC2, and cyclin B [47]. Moreover, ginkgetin decreased total Rb, the level of cyclin A/CDK1 or CDK2 complex, and the expression of cyclin B/CDK1 complex in HepG2 cells, leading to S phase arrest [49]. For further details regarding the mechanism of ginkgetin in blocking the tumor cell cycle, refer to Fig. 2A.

### Regulating programmed cell death

#### Inducing apoptosis

Ginkgetin has been reported to induce cell apoptosis in a variety of cancer cell lines, including breast cancer [41, 42], cervical cancer [45], colon cancer [47], hepatocellular carcinoma [49], kidney cancer [50], leukemia [51], lung cancer [52], osteosarcoma [58], ovarian cancer [59], and prostate cancer [38, 60], within the concentration range of 5 to 80 μM. For example, treatment with ginkgetin at a concentration of 20 μM for 24 h resulted in apoptosis rates of 49.1 and 32.4% in ovarian cancer cells SKOV3 and A2780, respectively [59]. Ginkgetin with a concentration of 20 to 80 μM induced the apoptotic rates from 8.5 to 33.5% in breast cancer cells MCF-7 [42].

On the one hand, ginkgetin triggered the intrinsic apoptosis pathway, demonstrated by increased mitochondrial outer membrane permeabilization (MOMP) and the release of Cytochrome *c* into the cytoplasm, which activated the caspase cascade and upregulated cleaved caspase-3, caspase-9, and PARP, ultimately leading to apoptosis [38, 42, 44, 47, 49, 52, 58–60]. Ginkgetin modulated the MOMP through regulating the activity of Bcl-2 protein family, including upregulating pro-apoptotic members such as Bax and downregulating anti-apoptotic members such as Bcl-2 and Bcl-xL [38, 42, 44, 49, 52, 58–60]. Moreover, ginkgetin mediated the activation of caspase cascade by the intracellular reactive oxygen species generated possibly through auto-oxidation of this biflavone, leading to apoptosis in OVCAR-3 cells [45]. On the other hand, ginkgetin induced extrinsic apoptosis in leukemia K562 cells by increasing levels of TNF-α, hereby inducing death receptor-mediated extrinsic apoptosis [51].

Ginkgetin induced apoptosis in cancer cells by regulating multiple intracellular pathways including JAK2/STAT3 [38, 50, 52, 59, 65], MAPK pathways [42, 59], the ER signaling pathway [41], and miRNA-122-5p/GALNT10 axis [44]. Ginkgetin reduced the protein levels of p-JAK2, p-STAT3, p-JAK1, and src, blocking the JAK2/STAT3 pathway [38, 50, 52, 59]. Specifically, ginkgetin upregulated both protein and mRNA levels of SHP-1 in A549 cells and PTEN in FaDu cells, which are involved in regulating STAT3 signaling, thus suppressing nuclear translocation of STAT3 and STAT3 phosphorylation at tyrosine 705 [52]. Furthermore, ginkgetin had a dual regulatory effect on MAPK signaling. Ginkgetin exhibited anti-tumor activity in ovarian cancer cells via inhibiting the MAPK pathways, and SIRT1 protein [59]. Conversely, ginkgetin increased the expression of MAPKs, including p-p38, p-JNK, as well as p-ERK1/2 in breast cancer cells MCF-7 [42]. These proteins negatively regulate breast cancer growth, which has been proven by the ability of p-JNK and p-ERK1/2 inhibitors to prevent drug-induced apoptosis in vitro [66–68]. In breast cancer cells MCF-7 and T-47D, ginkgetin impaired the ER signaling pathway via downregulating the expression of ER‑α, which directly downregulated the expression of PFKFB3, cyclin D1, and survivin [41]. Moreover, ginkgetin treatment upregulated miR-122-5p, leading to specific binding to the GALNT10 3′-untranslated regions, which ultimately reduced Smad3 mRNA and protein levels in MDA-MB-231 breast cancer cells [44]. Figure 2B illustrates these mechanisms. On balance, the activation of cancer cell apoptosis by ginkgetin fully illustrates its ability to effectively inhibit tumor proliferation and development.

#### Regulating autophagy

Autophagy is a key homeostatic pathway that facilitates the degradation and recycling of cellular material [69]. The role of autophagy in controlling cll death renders it a viable candidate for cancer therapy [70, 71]. Ginkgetin induced autophagic cell death in non-small cell lung cancer (NSCLC) A549 cells, which was inhibited by the autophagosome formation blocker 3-methyladenine; ginkgetin treatment also increased autophagy marker LC3 I/II and decreased p62 [54]. Further research revealed that ginkgetin had potential binding affinity to p62, disrupting the p62-mTORC1 complex and increasing ATG7, subsequently enhancing autophagosome-lysosome fusion and lysosome activity [54]. Additional details can be seen in Fig. 2C. While studies exploring ginkgetin's regulation of autophagy in cancer remain limited, evidence confirmed its efficacy in mitigating cerebral ischemia/reperfusion injury via suppression of NF-κB/p53-mediated autophagy [72], and in ameliorating diabetic nephropathy progression through AMPK/mTOR-dependent autophagy activation, which attenuates high glucose-induced mesangial cell dysfunction [73]. These observations of ginkgetin's ability to modulate autophagy through diverse mechanisms highlight the importance of investigating its autophagy-related anticancer effects. However, autophagy has a dual role in cancer, and its ability to promote or inhibit cancer depends on tumor staging, biology, and the surrounding microenvironment [74]. Autophagy promotes cell death by degrading damaged organelles and proteins, enhancing chemotherapy/radiotherapy efficacy [71, 75]; yet under stress (nutrient deprivation or therapy), it acts as a pro-survival mechanism inducing treatment resistance [76, 77]. In summary, it is necessary to further explore and elucidate how ginkgetin targets and regulates autophagy to achieve precision therapy.

#### Promoting ferroptosis

Ferroptosis, an iron-mediated regulated cell death process, is initiated by lipid peroxidation and commonly induced through inhibition of solute carrier family 7 member 11 (SLC7A11) and glutathione peroxidase 4 (GPX4) [78, 79]. Ginkgetin synergized with cisplatin to increase ferroptosis in NSCLC cells, which was confirmed by the decreased expression of SLC7A11 and GPX4, and a decreased reduced glutathione/oxidized glutathione disulfide (GSH/GSSG) ratio [61]. This was because ginkgetin suppressed the Nrf2/HO-1 antioxidant system, which is usually promoted by cancer cells to protect themselves from ferroptosis-induced cell death [61]. Similarly, when breast cancer cells generated radioresistance, ginkgetin promoted ferroptosis in 4T1 cells after radiotherapy by suppressing the Nrf2/HO-1 axis activity, elevating intracellular levels of reactive oxygen species (ROS) and ferrous ions, accelerating GSH depletion, and reducing GPX4 expression [43]. Ginkgetin downregulated GPX4 may be due to its superior binding affinity to GPX4 compared to ferroptosis inducer RSL3 according to computational screening, suggesting that ginkgetin can be used as a GPX4 inhibitor to promote ferroptosis, though requiring experimental validation [80]. Additionally, ginkgetin binds to and activates TFEB, which facilitates the formation of a TFEB-TRIM25 complex [81]. This interaction enhances TRIM25-mediated ubiquitination of GPX4 by promoting TRIM25-GPX4 binding while disrupting the USP5-GPX4 interaction, ultimately leading to GPX4 degradation in lung adenocarcinoma cells [81]. Moreover, p53 also regulates ferroptosis through either a transcriptional or posttranslational mechanism [82]. In HCT-116 cells, treatment with 20 μM ginkgetin for 24 h enhanced p53 transcriptional activity and caused ferroptosis, as evidenced by a significant increase in iron content in cells and a significantly reduced GSH content and the strongest reversal effect caused by Fer-1 [48]. Ginkgetin has been proven to cause oxidative stress in some cancer cells [48], thus it has great potential for regulating ferroptosis, as this coincides with the disruption of the balance between the production and consumption of intracellular lipid reactive oxygen species, which is the core mechanism of ferroptosis. Figure 2D illustrates the partial mechanisms of ginkgetin-induced ferroptosis in cancer cells. In general, further investigation into the role of ginkgetin in regulating ferroptosis and its implications for cancer therapy is valuable.

### Inhibiting angiogenesis

Angiogenesis, the development of new blood vessels, is a highly regulated and intricate process controlled by both stimulatory and inhibitory factors, enabling tumor cells to obtain significantly more oxygen and nutrients than ordinary cells to support their rapid growth [83]. Thus, angiogenesis plays a vital role in tumor growth, infiltration, and metastasis. Vascular endothelial growth factor (VEGF) is the key angiogenic factor that contributes to the formation of disorganized and primitive vasculature in various tumor tissues [84]. Molecular docking, surface plasmon resonance (SPR), and immunoprecipitation experiments have demonstrated the direct binding between ginkgetin and VEGF, indicating that ginkgetin could inhibit VEGF-mediated angiogenesis and endothelial cell proliferation [62]. In HUVEC cells, this intervention effectively blocked the phosphorylation cascade involving vascular endothelial growth factor receptor 2 (VEGFR2) and its downstream effectors (Akt, eNOS, and ERK), while concurrently suppressing MMP-2/9 expression [62]. This mechanism is depicted in Fig. 2E. Above all, ginkgetin demonstrates the ability to effectively inhibit vascular mimicry, which further contributes to the suppression of tumor growth and metastasis.

### Inhibiting migration and invasion

Since metastasis dominates solid tumor mortality, deciphering drugs' anti-metastatic mechanisms remains a pressing research priority [85]. Wound-healing and Transwell assays demonstrated that ginkgetin within the range of 2.5 to 10 μM has shown the ability to reduce the migration and invasion of various cancer cells in vitro experiments, including A549 and H1299 lung cancer cells [53, 55], A2780, SK-OV-3, and CP70 ovarian cancer cells [59], suggesting its anti-cancer potential in resisting metastasis.

Matrix metalloproteinases (MMPs) play a vital role in metastasis in several cancers [86]. After treatment with ginkgetin, the protein levels of MMP2 and MMP9 significantly decreased [59]. The anti-migration and anti-invasion effects of ginkgetin were mediated through the inhibition of oncogenic JAK2/STAT3 and MAPKs/ERK pathways in ovarian cancer cells [53, 59]. Thus, ginkgetin inhibits the JAK2/STAT3 and MAPKs/ERK pathways in tumor cells, not only leading to apoptosis but also suppressing migration and invasion. Similarly, ginkgetin inhibited EGF-induced phosphorylation of FAK, STAT3, and AKT, effectively blocking the FAK/STAT3/AKT signaling axis and suppressed EGF-induced proliferation and migration of A549 and H1299 cells [55]. Additionally, ginkgetin-mediated inhibition of the miR-122-5p/GALNT10/Smad3 signaling axis significantly attenuated the migratory capacity of breast cancer MDA-MB-231 cells [44]. Moreover, in human lung adenocarcinoma models, ginkgetin suppressed metastasis by inhibiting epithelial-to-mesenchymal transition (EMT), the fundamental process driving acquisition of migratory and invasive capabilities [87], with significantly reduced EMT markers N-cadherin and vimentin levels [53]. Further research has confirmed that ginkgetin treatment disrupted the core Akt/GSK-3β/Snail EMT axis, downregulating p-Akt(Ser473), GSK-3β, Snail, and c-Myc while upregulating p-GSK-3β [53]. This pathway suppression was structurally validated by ginkgetin's strong binding to AKT1, GSK3β, and CTNNB1 (β-catenin) [53], directly inhibiting Snail regulation through both Akt/GSK-3β/Snail and Wnt/β-catenin pathways [88, 89]. Developing effective strategies for treating metastatic tumors is more challenging than targeting primary tumors. Ginkgetin's ability to inhibit tumor metastasis highlights its potential as a therapeutic agent and offers a promising strategy for treating metastatic tumors. Further studies on various tumor types focusing on metastasis are needed to validate these findings. A schematic representation of this mechanism is graphically summarized in Fig. 2F.

## Potential targets

The identification of direct binding targets in natural products has always been an important topic explored by researchers [90]. Some existing studies identified direct targets of ginkgetin, among which VEGF, p62 and TFEB are likely confirmed targets of ginkgetin in colon cancer, lung cancer and lung adenocarcinoma, respectively [54, 62, 81]. Molecular docking, SPR, and immunoprecipitation (IP) have revealed that ginkgetin directly targeted VEGF, thereby suppressing the angiogenic properties of VEGF [62]. Similarly, molecular docking assays and ultrafiltration-based affinity assay showed that ginkgetin can bind to p62, consequently suppressing the formation of the p62-mTORC1 complex and leading to autophagy [54]. In addition, SPR and microscale thermophoresis (MST) assays confirmed ginkgetin directly binds to TFEB, inducing its nuclear translocation and subsequent lysosomal activation and GPX4 degradation, ultimately triggering ferroptosis [81]. Since ginkgetin is likely to be multitargeted, further investigations are needed to demonstrate its enormous therapeutic potential in treating various kinds of cancers by affecting different signaling pathways.

Beyond the validated direct targets of ginkgetin, computational prediction techniques have identified additional potential targets of ginkgetin in recent studies, though their binding affinities and functional relevance await experimental confirmation. Virtual screening based on molecular docking, molecular dynamics (MD) simulation, and molecular mechanics/generalized Born surface area (MM/GBSA) free energy calculations confirmed that ginkgetin has a good binding affinity for c-Myc G-Quadruplex (G4), exerting anti-proliferative effects via G4-stabilization-induced transcriptional inhibition of the c-Myc oncogene [57]. Moreover, silico targeting and MD simulation indicated the interaction of ginkgetin with CDK2 through π-alkyl and hydrogen bonds, thus arresting the hepatocellular carcinoma HepG2 cells in the G1/S phase [28]. Furthermore, virtual screening combined with MD simulations showed minimal fluctuations in the binding mode between ginkgetin and Hsp90, suggesting that ginkgetin may be an effective Hsp90 inhibitor [91]. Integrated computational analyses nominated AKT1, CTNNB1, and GSK3β as potential therapeutic targets of ginkgetin in lung cancer, consistent with observed reductions in p-AKT and total GSK-3β alongside elevated p-GSK-3β levels post-treatment, thus experimental validation remains essential to determine whether modulation occurs via direct binding [53]. Nevertheless, although virtual computing techniques provide valuable preliminary insights, these in silico approaches alone are insufficient to fully characterize biomolecular interactions. Experimental validation through in vitro binding assays and in vivo functional studies is essential to confirm the predicted binding affinities and elucidate the pharmacological relevance of these interactions in a biological context.

## Future research directions

### Optimizing ginkgetin’s druggability

**Fig.3:**
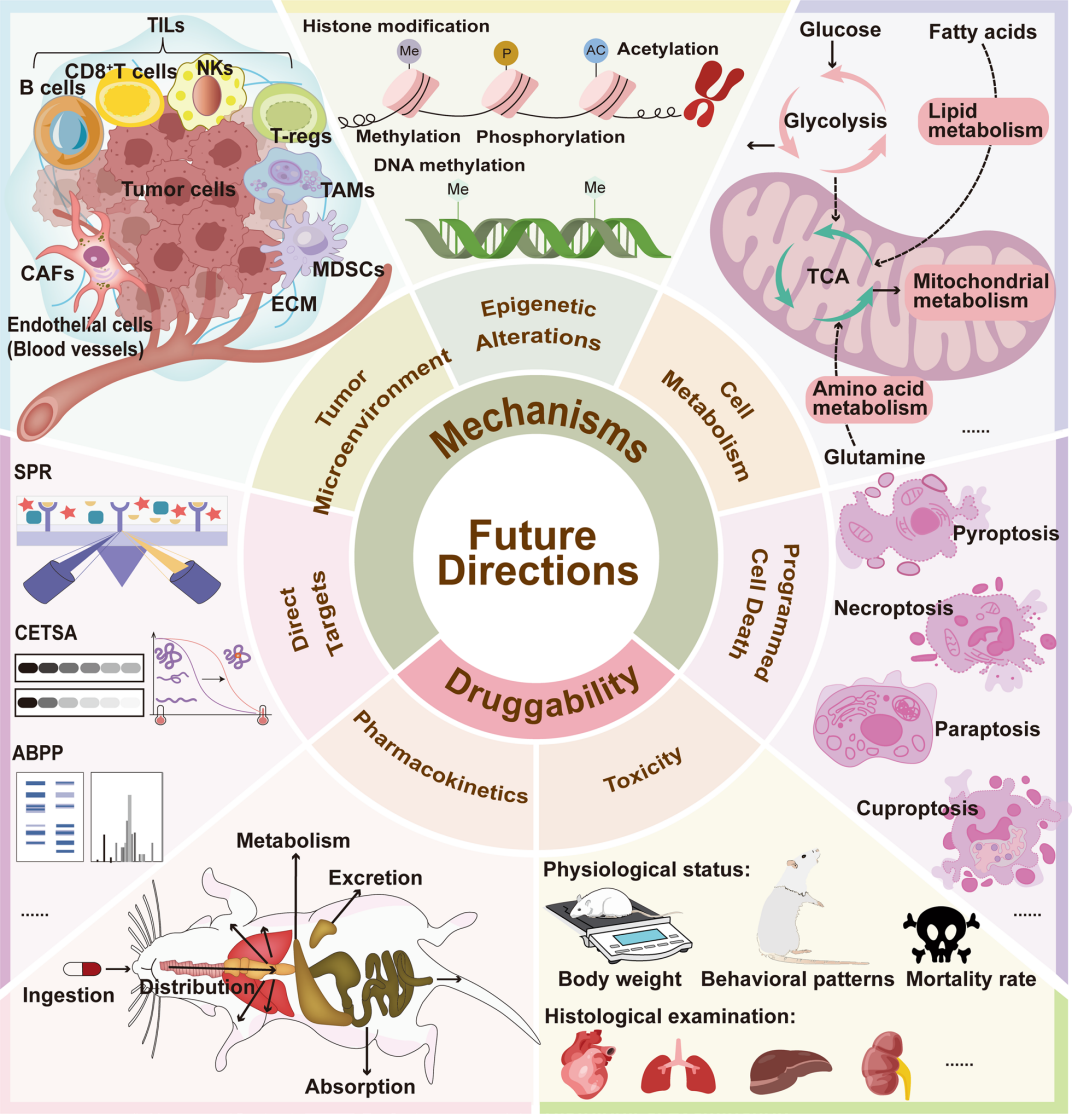
Schematic diagram of future directions in studying ginkgetin. To advance the clinical translation of ginkgetin for cancer therapy, future studies should prioritize pharmacokinetics, toxicity profiling, and deeper exploration of its anticancer mechanisms. Further mechanistic investigations should explore whether ginkgetin modulates additional forms of programmed cell death, influences cancer cell metabolic reprogramming and epigenetic modifications, and remodels the tumor microenvironment. Additionally, identifying direct targets of ginkgetin through advanced techniques is critical to elucidate its mode of action

All these data and studies discussed in this review illustrate that ginkgetin has significant anti-cancer effects by inhibiting proliferation across various cancer cell types and suppressing multiple tumor progression in xenograft nude mouse models. The IC_50_ values of ginkgetin treatment ranged from 0.58 to 150 μM, which varied due to differences in cell type, treatment time, number of plated cells, and treatment method. For animal experiments, the concentration of administration ranged from 10 to 100 mg/kg, owing to different types of tumors and administration methods. In addition to standalone use, ginkgetin has synergistic effects with other drugs by enhancing drug efficacy and alleviating side effects. Therefore, ginkgetin demonstrates potential as an anti-cancer therapeutic agent or as a sensitizer to enhance efficacy or reduce side effects when combined with other chemotherapeutic drugs. Systematic chemical modifications based on its core structure represent a viable approach for developing novel agents with improved potency and selectivity. However, the lack of clinical data limits ginkgetin’s drug-likeness. In current clinical practice, ginkgetin is primarily administered within herbal preparations or GBLs extracts, where therapeutic effects are promoted through multi-component interactions. No clinical trials have yet employed ginkgetin as a standalone therapeutic agent. To advance its drug development potential and clinical translation, critical research gaps remain to be addressed in the following areas.The relevant content can be found in Fig.3: Druggability.

Firstly, current pharmacokinetic research on ginkgetin remains limited, lacking reliable research data on the absorption, distribution, metabolism, and excretion (ADME) of ginkgetin in vivo. This knowledge deficit likely stems from inherent physicochemical challenges including: low natural abundance in plant matrices, low molecular polarity, poor aqueous solubility, slow dissolution kinetics, and consequently low bioavailability—all of which collectively impede comprehensive ADME profiling. The inherently poor aqueous solubility of ginkgetin severely compromises their oral bioavailability [92]. Consequently, improving the bioavailability and water solubility of ginkgetin is an urgent issue that needs to be addressed. In addition to considering administration routes such as intraperitoneal or intravenous injection, improvements can be made through innovative dosage form design and advanced drug delivery platforms, such as particularly exosome- and extracellular vesicle-mediated delivery systems for ginkgetin [27, 93]. While current research on ginkgetin-specific delivery remains limited, valuable insights may be drawn from extensive existing studies on flavonoid drug delivery in GBLs [94, 95]. Furthermore, it is worth mentioning that research has shown that ginkgetin exhibited significant inhibition activity towards CYP3A4, which is a pivotal enzyme in the metabolic processing of many commonly used drugs, and the IC_50_ value was evaluated as 0.106 ± 0.004 μM [96]. Hence, investigating ginkgetin-mediated inhibition of clinically relevant CYP3A4 substrates (e.g., tamoxifen, gefitinib) is essential to predict herb-drug interactions in oncology.

Analogously, comprehensive toxicity evaluation of ginkgetin remains understudied, primarily because its safety profile has been inferred from the historical use of GBLs extracts containing this compound. Meanwhile, ginkgetin did not show toxicity in animal experiments, as evidenced by no change in body weight and overall health status between the experimental group and the control group. However, ginkgetin may have potential liver and kidney toxicity according to some studies, highlighting the need for further investigation into its safety profile. In in vivo experiments, after intragastrical administration of ginkgetin at 20 mg/kg for consecutive 7 days in mice, the activity of alkaline phosphatase was significantly increased and widespread hydropic degeneration of hepatocytes was observed in ginkgetin -treated mice [97]. Moreover, ginkgetin induced acute kidney injury in treated mice and the main pathological lesions were confirmed in the tubule, glomeruli, and interstitium injuries [97]. Additionally, ginkgetin displayed potent Human UDP-glucuronosyltransferase 1A1 (hUGT1A1) inhibition in HeLa-UGT1A1 cells (Hela cells overexpressing hUGT1A1), which is one of the most important conjugative enzymes in the human body and plays a crucial role in the metabolic detoxification of endogenous toxicants (e.g., bilirubin) and a variety of clinical drugs (e.g., SN38) [98]. Therefore, to contribute to its rational and effective utilization, comprehensive safety evaluations through acute and subacute toxicity studies are imperative [99, 100]. Observing the physiological status of mice during administration, such as body weight, behavioral patterns, and mortality rate, subsequently, performing histological examination of organs such as the heart, lungs, liver, and kidneys is essential.

### Deciphering ginkgetin’s anticancer mechanisms

According to the above studies, ginkgetin reduces cancer cell proliferation by blocking the cell cycle and inducing different cell death mechanisms, including apoptosis, autophagy, and ferroptosis. Ginkgetin also suppresses metastatic dissemination through impairment of tumor cell motility and invasive capacity. Finally, ginkgetin prevents angiogenesis to impede tumor growth and metastasis. Ginkgetin exerts its antitumor effects by modulating multiple oncogenic signaling cascades in tumor cells, notably the JAK/STAT, Wnt/β-catenin, AKT/GSK-3β, MAPK and ER pathway. Furthermore, ginkgetin modulates the expression of microRNAs such as miR-122-5p and miR-34a, which play pivotal roles in cancer pathogenesis by either cleaving target mRNAs or regulating the transcriptional activity of downstream genes. On the whole, ginkgetin exerts its anti-cancer effects through multiple mechanisms, as it can target multiple aspects of tumor cells and affect multiple processes in tumor development, which makes it uniquely potential for treating systemic diseases induced by multiple factors, such as cancer.

Except for these mechanisms revealed above, more potential anti-cancer mechanisms mediated by ginkgetin are worth further investigation. These aspects are illustrated in Fig.3: Mechanisms. Firstly, it is important to investigate whether ginkgetin can lead to more forms of programmed cell death in cancer cells, such as pyroptosis, necroptosis, paraptosis, cuproptosis, and so on. There have been numerous studies reporting the role of flavonoids in inducing pyroptosis, necroptosis, paraptosis, and cuproptosis of tumor cells [101, 102], so ginkgetin may exhibit similar effects, despite being overlooked in prior studies. Moreover, how ginkgetin acts on cancer cell metabolisms, including mitochondrial metabolism, glycolysis, lipid metabolism and amino acid metabolism still remains unclear. The crucial interplay between cancer cell metabolic reprogramming and its survival and proliferation has led to studying ginkgetin in the context of altered metabolism in cancer becoming a captivating and promising frontier. The regulatory effects of ginkgetin on the PI3K/Akt/mTOR signaling pathway and AMPK pathway demonstrate its potential in regulating tumor cell metabolism [37, 73, 103]. Last but not least, mechanistic dissection of ginkgetin-induced epigenetic alterations, encompassing histone modification (methylation/phosphorylation/acetylation) and DNA methylation [104, 105], is imperative for delineating its inhibiting roles in oncogenic transformation and malignant progression.

At present, research on ginkgetin is limited to its direct killing effect on tumor cells, ignoring its role in the tumor microenvironment (TME). TME is a multicellular ecosystem made up of tumor cells, stromal constituents (including cancer-associated fibroblasts [CAFs] and endothelial cells), immune infiltrates (tumor-infiltrating lymphocytes [TILs], tumor-associated macrophages [TAMs], and myeloid-derived suppressor cells [MDSCs]), and acellular components such as the biomechanically remodeled extracellular matrix (ECM), that play a critical role in tumor progression and treatment resistance [106–108]. Nowadays, immunotherapy and targeting TME have been proven to be effective methods for treating various cancer [109–111]. Unfortunately, no direct research has explored the ability of ginkgetin to inhibit tumor growth through immune system regulation. Nevertheless, ginkgetin had been proven to have a regulatory effect on the immune system in many other diseases such as non-alcoholic steatohepatitis (NASH). Ginkgetin reprogramed macrophage polarization by shifting their functional state from pro-inflammatory (M1) to anti-inflammatory (M2) phenotypes [112]. Moreover, ginkgetin reduced inflammation related proteins such as PGE2 and TNF-α to exert its anti-inflammatory effects. Inflammation can affect the TME by inducing immune suppression, for example, PGE2 limits effector expansion of tumor infiltrating stem-like CD8^+^ T cells [113, 114]. Overall, ginkgetin has a great chance to exert anti-cancer functions by regulating the immune system, so its role in tumor immunotherapy and TME should be studied more extensively. In this context, more in vivo models such as patient-derived xenograft (PDX) models [115, 116] and humanized immune system mouse models [116] need to be applied since nude mice lack T cell immunity.

The currently confirmed direct anti-cancer targets of ginkgetin are VEGF, p62 and TFEB [54, 62, 81]. Identification of molecular targets for natural products serves as a critical nexus connecting natural product discovery with therapeutic validation and pharmacotherapeutic innovation [117]. With the development of chemical biology technology, more and more methods have been established and used for target identification of active ingredients in traditional Chinese medicine, such as surface plasmon resonance (SPR), cellular thermal shift assay (CETSA), and activity-based protein profiling (ABPP) [118, 119]. Furthermore, with the advancement of chemical biology and omics technologies, various target identification methods such as chemical proteomics, chemical genomics, multi-omics technologies, single-cell-based technologies, bioinformatics, and whole transcriptome tag analysis continue to emerge [120, 121]. In addition, artificial intelligence can also help predict the targets of natural products and shorten the screening time in the early stage [122]. In brief, the development of target identification technology promotes the determination of potential anti-cancer targets of ginkgetin, thereby promoting research on its therapeutic mechanisms and enhancing its druggability.

## Conclusion

In summary, ginkgetin exerts significant anti-cancer effects through various mechanisms. In the future, further research is needed to enhance its pharmacokinetics and improve its bioavailability to facilitate its translation into clinical applications. In addition, leveraging emerging technologies to gain a deeper understanding of the role of ginkgetin in the tumor immune microenvironment and to identify specific, direct molecular targets are directions worthy of further investigation.

## Data availability

The manuscript contains the full dataset and analytical outcomes derived from this investigation.

## Abbreviations

5-FU: 5-Fluorouracil
ABPP: Activity-based protein profiling
AKT: Protein kinase B
ATG7: Autophagy related 7
Bcl-2: Poly Adp-ribose polymerase
CAFs: Cancer-associated fibroblasts
CDK1: Cyclin dependent kinase 1
CDK2: Cyclin dependent kinase 2
CDKs: Cyclin-dependent kinases
CETSA: Cellular thermal shift assay
CTNNB1: Catenin beta 1
CYP3A4: Cytochrome P450 family 3 subfamily A member 4
ECM: Extracellular matrix
EMT: Epithelial-to-mesenchymal transition
eNOS: Endothelial nitric oxide synthases
ER: Estrogen receptor
ERK: Extracellular regulated protein kinases
G4: G-quadruplex
GALNT10: Polypeptide n-acetylgalactosaminyltransferase 10
GBLs: *Ginkgo biloba* Leaves
GPX4: Glutathione peroxidase 4
GSH/GSSG: Glutathione/oxidized glutathione disulfide
GSK-3β: Glycogen synthase kinase 3 beta
Hsp90: Heat shock protein 90
hUGT1A1: Human Udp-glucuronosyltransferase 1A1
*i.p.*: Intraperitoneal injections
IP: Immunoprecipitation
JAK1: Janus kinase 1
JAK2: Janus kinase 2
JNK: c-Jun n-terminal kinase
LC3: Microtubule-associated protein 1 light chain 3
MAPK: Mitogen-activated protein kinase
MD: Molecular dynamics
MDSCs: Myeloid-derived suppressor cells
miRNA34a: MicroRNA 34A
MM/GBSA: Molecular mechanics/generalized born surface area
MMPs: Matrix metalloproteinases
MOMP: Mitochondrial outer membrane permeabilization
MST: Microscale thermophoresis
NASH: Non-alcoholic steatohepatitis
NRF2/HO-1: Nuclear factor erythroid 2-related factor 2/heme oxygenase-1
NSCLC: Non-small cell lung cancer
*p.o.*: Oral administration
PARP: Poly Adp-ribose polymerase
PDX: Patient-derived xenograft
PFKFB3: 6-Phosphofructo-2-kinase/fructose-2,6-biphosphatase 3
PGE2: Prostaglandin E2
ROS: Reactive oxygen species
SIRT1: Sirtuin 1
SLC7A11: Solute carrier family 7 member 11
Smad: Drosophila mothers against decapentaplegic protein
SPR: Surface plasmon resonance
STAT3: Signal transducer and activator of transcription 3
TAMs: Tumor-associated macrophages
TFEB: Transcription factor Eb
TILs: Tumor-infiltrating lymphocytes
TME: Tumor microenvironment
TNF-α: Tumor necrosis factor
TRIM25: Tripartite motif containing 25
VEGF: Vascular endothelial growth factor
VEGFR2: Vascular endothelial growth factor receptor 2
